# Association between a single nucleotide polymorphism in the *R3HCC1* gene and irinotecan toxicity

**DOI:** 10.1002/cam4.5299

**Published:** 2022-10-29

**Authors:** Kou Kanesada, Ryouichi Tsunedomi, Shoichi Hazama, Hiroyuki Ogihara, Yoshihiko Hamamoto, Yoshitaro Shindo, Hiroto Matsui, Yukio Tokumitsu, Shin Yoshida, Michihisa Iida, Nobuaki Suzuki, Shigeru Takeda, Tatsuya Ioka, Hiroaki Nagano

**Affiliations:** ^1^ Department of Gastroenterological, Breast and Endocrine Surgery Yamaguchi University Graduate School of Medicine Ube Yamaguchi Japan; ^2^ Graduate School of Sciences and Technology for Innovation Yamaguchi University Ube Yamaguchi Japan; ^3^ Oncology Center Yamaguchi University Hospital Ube Yamaguchi Japan

**Keywords:** colorectal cancer, irinotecan, neutropenia, pancreatic cancer, R3HCC1

## Abstract

**Objective:**

Irinotecan is a useful anticancer drug for colorectal cancer treatment. UGT1A1*28 and *6 gene polymorphisms are known risk factors for irinotecan‐associated toxicity. However, severe adverse effects due to irinotecan have been observed even in patients who do not harbor UGT1A1*28 or *6. We investigated gene polymorphisms in the whole exome to identify useful biomarkers for irinotecan toxicity other than *UGT1A*.

**Methods:**

A total of 178 patients with metastatic colorectal cancer (mCRC) and 87 patients with pancreatic cancer were treated with FOLFIRI, FOLFOX, FOLFOXIRI, modified FOLFIRINOX, or gemcitabine plus nab‐paclitaxel. Genome‐wide screening was performed using whole‐exome sequencing (WES), and validation analysis was performed using qPCR with a hydrolysis probe.

**Results:**

Using WES after a doublet chemotherapy regimen comprising irinotecan and 5‐fluorouracil (*n* = 15), seven single nucleotide polymorphisms (SNPs) were identified as candidate biomarkers for irinotecan‐associated toxicity of neutropenia. Among the seven SNPs, an SNP in R3H domain and coiled‐coil containing 1 (*R3HCC1*; c.919G > A, rs2272761) showed a significant association with neutropenia (>grade 3) after doublet chemotherapy. Patients receiving irinotecan including triplet chemotherapy, FOLFOXIRI for mCRC (*n* = 23) or modified FOLFIRINOX for pancreatic cancer (*n* = 40), also showed significant linear trends between *R3HCC1* polymorphism and neutropenia (*p* = 0.017 and 0.046, respectively). No significant association was observed in patients treated with irinotecan‐free regimens, FOLFOX for mCRC (*n* = 66), and gemcitabine plus nab‐paclitaxel for pancreatic cancer (*n* = 47).

**Conclusion:**

Thus, an SNP in the *R3HCC1* gene may be a useful biomarker for the toxicity of irinotecan‐containing chemotherapy for mCRC and pancreatic cancer.

## Graphical abstract

We utilized whole‐exome sequencing and PCR validation with colorectal and pancreatic cancer patient data in order to identify novel biomarkers that may be predictive of adverse effects associated with irinotecan‐containing chemotherapy, such as FOLFIRI, FOLFOXIRI, and modified FOLFIRINOX therapies. Among the variations identified, we found that a single nucleotide polymorphism in R3HCC1 showed a significant linear relationship with toxicity and may explain the adverse effects in patients who did not harbor known susceptibility‐conferring polymorphisms in UGT1A. Our findings offer a new target to test for when deciding on chemotherapy regimen choice and dosages and improves our approach of precision medicine in colorectal and pancreatic cancer patients.

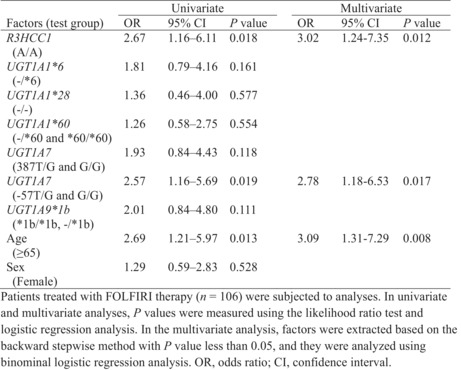

## 1 INTRODUCTION

Colorectal cancer (CRC) is one of the leading causes of cancer‐related deaths, and over a million new cases are diagnosed per year globally.^1^, ^2^ Recently, new regimens combining chemotherapy and molecular targeted agents for metastatic colorectal cancer (mCRC) have been reported to improve progression‐free survival (PFS) and overall survival (OS).^3^, ^4^, ^5^, ^6^ The standard treatment for mCRC usually consists of chemotherapy with 5‐fluorouracil or capecitabine in combination with oxaliplatin or irinotecan and targeted agents such as bevacizumab, cetuximab, and panitumumab.^7^, ^8^, ^9^, ^10^ The most commonly used chemotherapy regimens are 5‐fluorouracil with leucovorin plus oxaliplatin (FOLFOX), capecitabine plus oxaliplatin (CAPOX), and 5‐fluorouracil with leucovorin plus irinotecan (FOLFIRI).

For the management of mCRC, FOLFIRI therapy is a useful tool.^11^, ^12^, ^13^, ^14^, ^15^, ^16^ Irinotecan‐containing regimens contribute to prolonged survival, but severe neutropenia occurs in 20%–45% of mCRC patients treated with irinotecan.^5^, ^12^, ^14^ In recent years, 5‐fluorouracil with leucovorin, oxaliplatin, and irinotecan (FOLFOXIRI) therapy has also been used as a powerful chemotherapy for mCRC.^17^, ^18^, ^19^ It has shown efficacy in mCRC, but is associated with a high frequency of toxicity, including severe neutropenia, similar to FOLFIRI therapy.^20^ Although 5‐fluorouracil with leucovorin, irinotecan, and oxaliplatin (FOLFIRINOX) therapy, like FOLFOXIRI therapy, has been shown to be effective as a triplet chemotherapy for poor prognostic pancreatic cancer, a high frequency of severe neutropenia has also been observed.^21^, ^22^

Polymorphisms in the *UGT1A* gene, which encodes the hepatic uridine diphosphate‐glucuronosyltransferase (UGT) 1A enzyme, are well‐known risk factors associated with irinotecan toxicity. Carboxylesterases catabolize irinotecan to 7‐ethyl‐10‐hydroxycamptothecin (SN‐38), which serves as a potent topoisomerase I inhibitor.^23^, ^24^ SN‐38 is further catabolized to the inactive SN‐38 glucuronide (SN‐38G) by the liver UGT1A enzyme, which is then excreted in bile.^25^ However, patients homozygous for UGT1A1*28 or *6 or compound heterozygous for UGT1A1*28 and *6 have been reported to have a high incidence of irinotecan‐related toxicity (neutropenia, diarrhea, etc.).^26^ Therefore, in 2005, the US Food and Drug Administration revised the Dosage and Administration section of the labeling of CPT‐11, recommending that reduction in the starting dose of the drug should be considered for patients homozygous for UGT1A1*28. Irinotecan dosage decisions based on UGT1A1*28 and *6 presence are routinely made in Japan, and a reduced dose of irinotecan is recommended for the aforementioned patients because UGT1A1*6 is relatively common in Asians, while UGT1A1*28 is less frequent in Asians (0.16) than in Caucasians (0.39).^27^

Interestingly, the toxicity and tumor response to FOLFIRI also correlate with *UGT1A* variants—*UGT1A1*, *UGT1A7*, and *UGT1A9*—and haplotypes including these variants.^28^, ^29^, ^30^, ^31^, ^32^, ^33^ However, there are reports that patients without UGT1A1*28 or *6 still show severe irinotecan toxicity.^14^ Therefore, identifying a distinct biomarker other than *UGT1A1* would contribute to a more precise anticancer therapy when used in combination with *UGT1A1*.

In this study, to explore biomarkers other than *UGT1A1* for irinotecan toxicity, genome‐wide screening was performed in patients who received FOLFIRI therapy. The identified candidate biomarker single nucleotide polymorphisms (SNPs) were tested with irinotecan‐containing triplet chemotherapies (FOLFOXIRI and modified FOLFIRINOX) and irinotecan‐free chemotherapies (FOLFOX and gemcitabine plus nab‐paclitaxel).

## 2 MATERIALS AND METHODS

### 2.1 Patients

In this study, 178 patients with mCRC and 87 with pancreatic cancer were examined for associations between genotypes and irinotecan toxicity (Table 1). For colorectal cancer, this study was performed as an ancillary investigation and data were collected from two prospective studies on FOLFIRI therapy as first and second line treatments for patients with mCRC.^34^, ^35^ Consecutive patients who received FOLFIRI for mCRC as a second‐line regimen (from April 2009 to October 2021), and patients who received FOLFOXIRI therapy for mCRC (from May 2015 to August 2021) at the Department of Gastroenterological Surgery, Yamaguchi University Graduate School of Medicine, Japan, were enrolled in this study. Among the consecutive patients, data were retrospectively collected from those who received FOLFIRI therapy as a second‐line regimen, but were both retrospectively (*n* = 17) and prospectively (*n* = 6) collected from those who received FOFOXIRI therapy. As a control group, data of patients who received FOLFOX therapy as first‐line therapy prior to second‐line irinotecan‐containing therapy and patients who received FOLFOX plus vaccine therapy were collected. For pancreatic cancer, data were collected from consecutive patients (from July 2015 to May 2021) who received modified FOLFIRINOX therapy and gemcitabine plus nab‐paclitaxel for pancreatic cancer as a case and a control, respectively, at the aforementioned institute. Data from patients who received modified FOLFIRINOX were collected retrospectively (*n* = 37) and prospectively (*n* = 3), and those from patients who received gemcitabine plus nab‐paclitaxel were also collected retrospectively (*n* = 45) and prospectively (*n* = 2).

**TABLE 1:** Characteristics of study patients

	Treatment regimens				
	Colorectal cancer			Pancreatic cancer	
Clinical features and genotypes	FOLFIRI (*n* = 106)	FOLFOXIRI (*n* = 23)	FOLFOX^a^ (*n* = 66)	mFOLFIRINOX (*n* = 40)	GEM + nab‐PTX (*n* = 47)
Toxicity^b^					
Yes	44	12	14	21	23
No	62	11	52	19	24
Lines of treatment					
1st line	35	23	66	33	41
2nd line	71	0	0	7	6
Sex					
Male	64	15	33	24	27
Female	42	8	33	16	20
Age					
<65	55	13	31	11	15
≥65	51	10	35	29	32
UGT1A1*6					
−/−	73	19	20	29	27
−/*6	33	4	6	11	17
*6/*6	0	0	1	0	3
UGT1A1*28					
−/−	87	20	16	32	29
−/*28	17	3	9	8	10
*28/*28	0	0	0	0	1

Abbreviations: GEM, gemcitabine; nab‐PTX, nab‐paclitaxel.

^a^ Patients who received mFOLFOX6 therapy as first‐line therapy prior to second‐line irinotecan‐containing therapy (*n* = 22) and patients who received mFOLFOX6 plus vaccine therapy (*n* = 44) were included. In the FOLFOX group, there were missing values for *UGT1A1* genotypes.

^b^ Toxicity: Yes, neutropenia greater than grade 3 during the entire course of therapy; No, neutropenia grade 0–2 during the entire course.

In this study, we defined patients who exhibited neutropenia greater than grade 3 during the entire course of therapy as experiencing irinotecan toxicity. Patients who exhibited grade 0–2 neutropenia during the entire course were defined as no toxicity group. The study protocols were approved by the Institutional Review Board of Yamaguchi University Graduate School of Medicine, and the study was conducted in accordance with the Declaration of Helsinki. Each patient provided written informed consent to participate in this study.

### 2.2 Chemotherapy regimen

The first‐ and second‐line FOLFIRI regimen used included irinotecan (100, 120, or 150 mg/m^2^) + 400 mg/m^2^ fluorouracil bolus, followed by 2400 mg/m^2^ fluorouracil continuous infusion for 46 h + 200 mg/m^2^ leucovorin every 2 weeks. Patients homozygous for UGT1A1*6 or *28 were excluded from this study because these patients received a lower starting dose of irinotecan (100 mg/m^2^). The FOLFOXIRI therapy used a 1‐h infusion of irinotecan (132, 150, or 165 mg/m^2^) + 2‐h infusion of oxaliplatin (85 mg/m^2^) and leucovorin (200 mg/m^2^), +48‐h continuous infusion of fluorouracil (2400, 2560, or 3200 mg/m^2^). Patients enrolled in a phase II study of five peptide vaccines in addition to oxaliplatin‐containing chemotherapy were used as the irinotecan‐free control population. mFOLFOX6 was used in addition to the vaccine, consisting of oxaliplatin (85 mg/m^2^) + 400 mg/m^2^ fluorouracil bolus + 46‐h continuous infusion of fluorouracil (2400 mg/m^2^) + leucovorin (200 mg/m^2^) every 2 weeks.^36^ The mFOLFOX6 regimen was also used as first‐line FOLFOX therapy in patients with colorectal cancer in this study.

Forty patients with pancreatic cancer were treated with modified FOLFIRINOX therapy every 2 weeks as follows: 2 h of intravenous (IV) injection infusion of oxaliplatin (85 mg/m^2^) + 2‐h of IV infusion of leucovorin (200 mg/m^2^) + 90 min of IV infusion of irinotecan (120 or 150 mg/m^2^) + 46 h of continuous IV infusion of fluorouracil (1920 or 2400 mg/m^2^). The remaining 47 patients were treated with nab‐paclitaxel (125 mg/m^2^) + gemcitabine (1000 mg/m^2^) on days 1, 8, and 15 every 28 days.

### 2.3 Whole‐exome sequencing (WES)

A conventional sodium iodide (NaI) method was used to extract genomic DNA from peripheral blood samples as previously described.^37^ DNA quantity was measured by both Qubit fluorometric quantitation (Thermo Fisher Scientific, Tokyo, Japan) and NanoDrop spectrophotometric quantitation (Thermo Fisher Scientific). DNA quality was examined using agarose gel electrophoresis. A total of 3 μg of DNA from each sample was used to prepare in vitro DNA libraries using the SureSelect Target Enrichment System (Agilent Technologies, Tokyo, Japan) with the SureSelectXT Reagent Kit (Agilent Technology) and the SureSelect Human ALL Exon V5 + UTRs (Agilent Technology), producing a total target size of 75 Mb. Sequencing of paired‐end fragments (100 bp × 2) was conducted on an Illumina HiSeq 2000 sequencing platform (Illumina, San Diego, CA, USA) at the Dragon Genomics Center at TaKaRa Bio (Mie, Japan).

### 2.4 WES data analysis

The obtained next‐generation sequencing data were subjected to read cleaning using Cutadapt (version 1.2.1)^38^ and cmpfastq_pe.pl software (http://compbio.brc.iop.kcl.ac.uk/software/cmpfastq_pe.php). After quality control, the filtered short reads were mapped to the reference genome (hg19) using BWA (version 0.7.12).^39^ The Genome Analysis Tool Kit (version 3.5)^40^ was used to perform local realignment and detect single nucleotide and insertion/deletion (InDel) polymorphisms. Furthermore, each detected variant was annotated with information such as the genome position and known functional effects using SnpEff (version 4.1 k).^41^ SnpEff, SIFT, and Polyphen‐2 were used to identify variants that were predicted to be damaging. A case–control association analysis was then conducted using PLINK (version 1.902b3w),^42^ utilizing the trend‐model (Cochran–Armitage test). Furthermore, the obtained variants were ranked by standardized differences based on the frequencies of the variant allele between the case and control, as shown below.

$$d=\frac{|{\hat{P}}_{t}-{\hat{P}}_{c}|}{\sqrt{\frac{{\hat{P}}_{t}\left(1-{\hat{P}}_{t}\right)+{\hat{P}}_{c}(1-{\hat{P}}_{c})}{2}}}.$$

### 2.5 DNA genotyping

Genomic DNA was extracted from peripheral blood samples using the NaI method^37^ and then subjected to TaqMan hydrolysis probe assays using a LightCycler 480 system (Roche Diagnostics, Tokyo, Japan) to determine the genotype. PCR amplification was carried out as follows: initial denaturation at 95°C for 10 min, followed by 55 cycles of PCR with denaturation at 95°C for 15 s, and annealing/extension for 1 min at 60°C. Primers and probes for UGT1A1*60 (c.‐3279 T > C, rs4124874), *UGT1A7* (c.387 T > G, rs17868323), *UGT1A7* (c.‐57 T > G, rs7586110), UGT1A9*1b (c.‐118 T_9_ > T_10_, rs35426722, also called UGT1A9*22), *APCDD1L* (c.186A > G, rs1980576), *R3HCC1* (c.919G > A, rs2272761), *OR51I2* (c.400A > G, rs12577167), *MKKS* (c.1549C > T, rs1547), *EDEM3* (c.2507 T > G, rs9425343), *CSMD2* (c.1733A > G, rs474474), and *GATA2* (c.490G > A, rs2335052) were purchased from Applied Biosystems (Tokyo, Japan). Genotyping of UGT1A1*6 (c.211G > A, rs4148323) and UGT1A1*28 (TA6 > TA7) was performed using the Invader assay (LSI Medience Corporation, Tokyo, Japan, or SRL, Inc., Tokyo, Japan).

### 2.6 Statistical analyses

The Cochran–Armitage trend test was used to examine the linearity of the relationship between each genotype and irinotecan toxicity. The Fisher's exact test was used to assess the relationship between toxicity and each variant and to calculate the odds ratios (OR). Logistic regression analysis was used to analyze gene polymorphisms and toxicity in univariate and multivariate analyses. *p* values were measured using the likelihood ratio test. In the multivariate analysis, factors were extracted using the backward stepwise method based on *p*. JMP Pro 14 software (SAS Institute, Cary, NC, USA) was used to perform the calculations. *p* < 0.05 was considered statistically significant.

## 3 RESULTS

### 3.1 Identification of irinotecan toxicity‐related SNPs using WES

To identify germ‐line mutations that could further explain susceptibility to irinotecan toxicity, we performed WES. Patients with no *UGT1A* variations who exhibited hematologic toxicity (grade 3) throughout the entire course of irinotecan therapy and patients bearing one of the *UGT1A* heterogeneous variations who exhibited severe toxicity (grade 4) to irinotecan in the first course of treatment comprised the case group (*n* = 10). Patients with no *UGT1A* variations and no severe toxicity (grade 0) comprised the control group (*n* = 5).

In the discovery phase, the 15 patients underwent WES analysis. The mean coverage of target regions for all cases was greater than 70×, with 97% covering at least 10×. The number of variants (both SNPs and InDels) from WES analysis was approximately 200 thousand in each patient. After WES followed by variant filtering, 110 variants with putative functional impact, such as resulting in amino acid substitutions in conserved sequences, were identified as candidates associated with the susceptibility to irinotecan toxicity. Furthermore, the 110 variants were ranked by standardized differences based on the frequencies of the variant allele between the case and control groups; the top 10 variants were selected for further analysis. All selected variants were SNPs. Among the 10 SNPs, rs1980576, rs2272761, and rs1547 showed the same allele frequencies as rs3946003, rs13530, and rs1545, respectively, in the 15 discovery cases. Seven SNPs (*APCDD1L* (c.186A > G, rs1980576), *R3HCC1* (c.919G > A, rs2272761), *OR51I2* (c.400A > G, rs12577167), *MKKS* (c.1549C > T, rs1547), *EDEM3* (c.2507 T > G, rs9425343), CSMD2 (c.1733A > G, rs474474), and *GATA2* (c.490G > A, rs2335052)) were eventually selected based on the results of WES (Table 2).

**TABLE 2:** Identification of SNPs associated with irinotecan‐related toxicity using whole‐exome sequencing

	Toxicity^b^				
Genotype	Yes	No	(% of Yes)	*p* value^a^	Standardized difference
*APCDD1L* (rs1980576)					
A/A	0	5	(0.0)	0.001	2.16
A/G	6	0	(100.0)		
G/G	4	0	(100.0)		
*R3HCC1* (rs2272761)					
G/G	0	4	(0.0)	0.003	1.98
G/A	2	0	(100.0)		
A/A	8	1	(88.9)		
*OR51I2* (rs12577167)					
A/A	9	0	(100.0)	0.001	1.82
A/G	1	3	(25.0)		
G/G	0	2	(0.0)		
*MKKS* (rs1547)					
C/C	0	5	(0.0)	0.001	1.73
C/T	8	0	(100.0)		
T/T	2	0	(100.0)		
*EDEM3* (rs9425343)					
T/T	7	0	(100.0)	0.002	1.71
T/G	3	2	(60.0)		
G/G	0	3	(0.0)		
*CSMD2* (rs474474)					
A/A	8	1	(88.9)	0.011	1.71
A/G	1	0	(100.0)		
G/G	1	4	(20.0)		
*GATA2* (rs2335052)					
G/G	7	0	(100.0)	0.002	1.71
G/A	3	2	(80.0)		
A/A	0	3	(0.0)		

^a^ Using the Cochran–Armitage trend test.

^b^ Toxicity: Yes, neutropenia greater than grade 3 during the entire course of therapy; No, neutropenia grade 0–2 during the entire course.

In addition to the *UGT1A* genotypes at six loci (UGT1A1*6 (c.211G > A), UGT1A1*28 (TA_6_ > TA_7_), UGT1A1*60 (c.‐3279 T > C), *UGT1A7* (c.387 T > G), *UGT1A7* (c.‐57 T > G), and UGT1A9*1b (c.‐118 T_9_ > T_10_)), we investigated the genotypes of 129 patients with mCRC at the seven SNPs chosen from WES. Subsequently, we evaluated the contribution of each genotype to the risk of irinotecan toxicity.

### 3.2 Validation phase of the association between screened SNPs and the toxicity of irinotecan

To validate the discovery phase, we statistically analyzed the relationship between the SNPs screened by WES and irinotecan toxicity in 91 patients who received FOLFIRI therapy, excluding the 15 patients who underwent WES. Among the seven SNPs, only *R3HCC1* (c.919G > A) showed a significant linear relationship with irinotecan‐related toxicity in the validation samples (*p* = 0.047) (Table 3). In the same cohort, among the six SNPs in *UGT1A, UGT1A7* (c.387 T > G), *UGT1A7* (c. − 57 T > G, linked with c.622 T > C), and UGT1A9*1b (c. − 118 T_9_ > T_10_) showed significant linear trends with irinotecan‐related toxicity (Table S1).

**TABLE 3:** Relationship between candidate SNPs and toxicity in validation samples

	Toxicity^c^				
Genotype	Yes	No	(% of Yes)	*p* value^a^	Odds ratio^b^
*APCDD1L* (rs1980576)					
A/A	10	24	(29.4)	0.182	A/A vs A/G, G/G
A/G	19	28	(40.4)		1.75 (*p* = 0.267)
G/G	5	5	(50.0)		
*R3HCC1* (rs2272761)					
G/G	0	4	(0.0)	0.047	G/G, G/A vs A/A
G/A	10	23	(30.3)		2.16 (*p* = 0.123)
A/A	24	30	(44.4)		
*OR51I2* (rs12577167)					
A/A	20	32	(38.5)	0.762	A/A vs A/G, G/G
A/G	11	19	(36.7)		0.90 (*p* = 0.830)
G/G	3	6	(33.3)		
*MKKS* (rs1547)					
C/C	14	27	(34.1)	0.307	C/C, C/T vs T/T
C/T	14	25	(35.9)		2.23 (*p* = 0.319)
T/T	6	5	(54.5)		
*EDEM3* (rs9425343)					
T/T	9	9	(50.0)	0.141	T/T vs T/G, G/G
T/G	19	32	(37.3)		0.52 (*p* = 0.278)
G/G	6	16	(27.3)		
*CSMD2* (rs474474)					
A/A	16	27	(37.2)	0.316	A/A, A/G vs G/G
A/G	13	29	(31.0)		9.66 (*p* = 0.026)
G/G	5	1	(83.3)		
*GATA2* (rs2335052)					
G/G	16	21	(43.2)	0.527	G/G vs G/A, A/A
G/A	14	30	(31.8))		0.66 (*p* = 0.382)
A/A	4	6	(40.0)		

^a^ Using the Cochran–Armitage trend test.

^b^ Using the Fisher's exact test.

^c^ Toxicity: Yes, neutropenia greater than grade 3 during the entire course of therapy; No, neutropenia grade 0–2 during the entire course.

Data from all 106 patients who received FOLFIRI therapy were analyzed using uni‐ and multi‐variate analyses. Selected SNPs (in *R3HCC1* and *UGT1A* as described above), age, and sex were used as factors in a binomial logistic regression analysis (Table 4). In the univariate analysis, *R3HCC1* (c.919A, OR = 2.67, 95% CI; 1.16–6.11, *p* = 0.018), *UGT1A7* (c.‐57G, OR = 2.57, 95% CI; 1.16–5.69, *p* = 0.019), and age (≥65 years‐old, OR = 2.69, 95% CI; 1.21–5.97, *p* = 0.013) showed higher OR (>2.5). In multivariate analysis, *R3HCC1* (c.877A, OR = 3.02, 95% CI; 1.24–7.35, *p* = 0.012), *UGT1A7* (c.‐57G, OR = 2.78, 95% CI; 1.18–6.53, *p* = 0.017), and age (≥65 years‐old, OR = 3.09, 95% CI; 1.31–7.29, *p* = 0.008) were identified as independent risk factors for susceptibility to irinotecan toxicity.

**TABLE 4:** Univariate and multivariate analyses results

	Univariate			Multivariate		
Factors (test group)	OR	95% CI	*p* value	OR	95% CI	*p* value
*R3HCC1* (A/A)	2.67	1.16–6.11	0.018	3.02	1.24–7.35	0.012
UGT1A1*6 (−/*6)	1.81	0.79–4.16	0.161			
UGT1A1*28 (−/−)	1.36	0.46–4.00	0.577			
UGT1A1*60 (−/*60 and *60/*60)	1.26	0.58–2.75	0.554			
*UGT1A7* (387 T/G and G/G)	1.93	0.84–4.43	0.118			
*UGT1A7* (−57 T/G and G/G)	2.57	1.16–5.69	0.019	2.78	1.18–6.53	0.017
UGT1A9*1b (*1b/*1b, −/*1b)	2.01	0.84–4.80	0.111			
Age (≥65)	2.69	1.21–5.97	0.013	3.09	1.31–7.29	0.008
Sex (Female)	1.29	0.59–2.83	0.528			

*Note*: Patients treated with FOLFIRI therapy (*n* = 106) were subjected to analyses. In univariate and multivariate analyses, *p* values were measured using the likelihood ratio test and logistic regression analysis. In the multivariate analysis, factors were extracted based on the backward stepwise method with *p* value less than 0.05, and they were analyzed using binominal logistic regression analysis.

Abbreviations: CI, confidence interval; OR, odds ratio.

### 3.3 Associations between the SNP in *R3HCC1* and the toxicity in patients with triplet chemotherapy

We examined the association between an SNP in *R3HCC1* and toxicity in 23 patients treated with FOLFOXIRI for colorectal cancer. There was a significant linear trend, similar to the FOLFIRI cases (*p* = 0.017), and an OR of 8.75 (c.919; G/G and G/A vs A/A, *p* = 0.036; Table 5). Interestingly, an SNP in *R3HCC1* was significantly associated with the toxicity of modified FOLFIRINOX as triplet chemotherapy in patients with pancreatic cancer (*n* = 40, *p* = 0.046 using the Cochran–Armitage trend test).

**TABLE 5:** Associations between the *R3HCC1* SNP and the toxicity in patients with irinotecan‐containing triplet chemotherapy

	Toxicity^c^				
*R3HCC1* (rs2272761)	Yes	No	(% of Yes)	*p* value^a^	Odds ratio^b^
FOLFOXIRI					
G/G	0	2	(0.0)	0.017	G/G, G/A vs A/A
G/A	2	5	(28.5)		8.75 (*p* = 0.036)
A/A	10	4	(71.4)		
mFOLFIRINOX					
G/G	1	2	(33.3)	0.046	G/G, G/A vs A/A
G/A	4	9	(30.8)		4.40 (*p* = 0.052)
A/A	16	8	(66.7)		

^a^ Using the Cochran–Armitage trend test.

^b^ Using the Fisher's exact test.

^c^ Toxicity: Yes, neutropenia greater than grade 3 during the entire course of therapy; No, neutropenia grade 0–2 during the entire course.

### 3.4 Associations between the SNP in *R3HCC1* and the toxicity in patients with irinotecan‐free regimens

We also examined the association between the *R3HCC1* SNP and susceptibility to irinotecan‐free toxicity. There was no significant association between an SNP in *R3HCC1* and patients receiving FOLFOX (*n* = 66) or gemcitabine plus nab‐paclitaxel (*n* = 47) therapies for colorectal or pancreatic cancer, respectively (Table 6).

**TABLE 6:** Associations between the *R3HCC1* SNP and toxicity in patients with irinotecan‐free regimens

	Toxicity^c^				
*R3HCC1* (rs2272761)	Yes	No	(% of Yes)	*p* value^a^	Odds ratio^b^
FOLFOX					
G/G	0	4	(0.0)	0.597	G/G, G/A vs A/A
G/A	7	23	(23.3)		1.08 (*p* = 1.000)
A/A	7	25	(21.9)		
GEM + nab‐PTX					
G/G	1	1	(50.0)	0.383	G/G, G/A vs A/A
G/A	7	10	(41.2)		1.83 (*p* = 0.380)
A/A	16	12	(57.1)		

Abbreviations: GEM, gemcitabine; nab‐PTX, nab‐paclitaxel.

^a^ Using the Cochran–Armitage trend test.

^b^ Using the Fisher's exact test.

^c^ Toxicity: Yes, neutropenia greater than grade 3 during the entire course of therapy; No, neutropenia grade 0–2 during the entire course.

## 4 DISCUSSION

In this study, we searched the whole exome for genetic polymorphisms that are associated with irinotecan‐related toxicity using WES because UGT1A1*28 and *6 are not sufficient to accurately predict susceptibility to severe irinotecan toxicity. WES is a high‐throughput technology that allows the sequencing of almost all protein‐coding genes of the human genome.^43^, ^44^ Our results showed that an SNP in *R3HCC1* (c.919G > A, rs2272761) was useful as a biomarker of hematotoxicity, such as severe neutropenia, in patients who received irinotecan‐containing doublet chemotherapy (i.e., FOLFIRI) for mCRC. *R3HCC1* may be a useful biomarker for irinotecan‐related toxicity in addition to *UGT1A* polymorphisms (Table 4). Interestingly, it was also found to be applicable to irinotecan‐containing triplet chemotherapy (i.e., FOLFOXIRI) for mCRC, and modified FOLFIRINOX therapy for pancreatic cancer (Table 5). However, it was not applicable to irinotecan‐free chemotherapy (i.e., FOLFOX) for mCRC or gemcitabine plus nab‐paclitaxel therapy for pancreatic cancer (Table 6). Screening for the *R3HCC1* gene polymorphism in addition to UGT1A1*28 and *6 prior to chemotherapy may improve the safety and efficacy of triplet chemotherapy.

**FIGURE 1:**
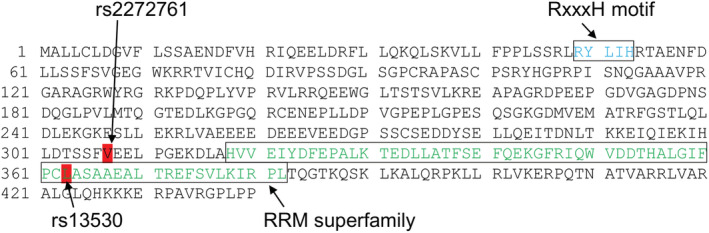
Amino acid sequence of human R3HCC1. Human R3HCC1 (NP_001129580.2) comprises 440 amino acids. Positions 49–53 (blue text) and 318–382 (green text) are the RxxxH motif and RNA recognition motif (RRM) superfamily, respectively. The single nucleotide polymorphisms rs2272761 (p.Val307Met) and rs13530 (p.Leu363Arg) are represented by red boxes.

The *R3HCC1* gene encodes R3H domain and coiled‐coil containing 1, which is thought to confer nucleic acid‐binding activity. The R3H domain binds to single‐stranded DNA and RNA in a sequence‐specific manner.^45^ For example, the R3H domain regulates Rbs1 (poly[A]mRNA‐binding protein) association with polymerase III.^46^ However, there are no reports, to the best of our knowledge, regarding the relationship between the R3H domain and chemotherapy‐induced neutropenia. The RNA recognition motif superfamily is located at amino acid sequence positions of 318–382 in R3HCC1 (NP_001129580.2). Interestingly, the SNP rs13530 (c.1088 T > G) corresponds to leucine363 of the R3HCC1 amino acid sequence (Figure 1). The SNP rs2272761 (c.919G > A) corresponding to valine307 of R3HCC1, showed perfect linkage to rs13530 in TaqMan SNP genotyping with 196 samples (data not shown). This finding suggests that *R3HCC1* polymorphism may affect the occurrence of neutropenia via its RNA‐binding function. However, the relationship between R3HCC1 and neutropenia remains unclear. Irinotecan is a pro‐drug, and its active form, SN‐38, is inactivated into SN‐38G. It is well known that UGT1A enzymes participate in inactivation of SN‐38 in the liver, causing adverse events in response to irinotecan. Metabolic processes including activation of the pro‐drug and uptake/efflux can also affect susceptibility to irinotecan.^47^, ^48^ Although pharmacokinetic analysis was not performed in this study, *R3HCC1* gene polymorphisms may be associated with altered SN‐38 pharmacokinetics.

With regard to the UGT1A1*6 polymorphisms, a meta‐analysis showed that patients homozygous for UGT1A1*6 had a high risk (OR = 2.95, vs genotype G/G) of severe neutropenia.^49^ Moreover, three previous studies revealed that patients homozygous for UGT1A1*28 showed a high risk (OR = 20.09,^50^ 6.04,^51^ and 8.61,^52^ vs genotype TA6/TA6) of severe neutropenia. Further, a significant association between UGT1A1*6 or *28 and severe neutropenia has been observed (OR = 19.82, vs haplotypes of G/G and TA6/TA6).^53^ In this study, uni‐ and multi‐variate analyses showed that *R3HCC1* had an OR of 2.67 and 3.02 for severe neutropenia in patients with mCRC, respectively (Table 4). Among patients receiving irinotecan‐containing therapy, the OR of the variant to the reference homozygous for *R3HCC1* (c.919A/A vs G/G) was 15.87. The OR of *R3HCC1* seems to be comparable to that of *UGT1A1* polymorphisms.

There are differences between Caucasian and Asian populations in their frequencies of *UGT1A* variants, and UGT1A1*6 and UGT1A1*28 are reportedly strongly associated with severe neutropenia, especially among Asian and Caucasian patients, respectively. According to the International HapMap Project, the frequency of the A allele of *R3HCC1* (rs2272761, c.919G > A), which is associated with a high risk of irinotecan toxicity, tends to be higher in Asian patients, such as Japanese (0.74) and Chinese (0.92), than in Europeans (0.58). Similarly, the allele frequency of the A allele was 0.75 in our 282 Japanese patients. In this study, the frequencies of the A allele of *R3HCC1* in patients receiving irinotecan‐containing therapy were 0.87 (>grade 3 neutropenia) and 0.67 (grade 0–2 neutropenia), while those in patients receiving irinotecan‐free chemotherapy were 0.67 (>grade 3 neutropenia) and 0.71 (grade 0–2 neutropenia). Similar to the UGT1A1*6 gene polymorphism, the *R3HCC1* gene polymorphism may be a risk factor for irinotecan toxicity that shows increased frequency in Asians. However, due to our limited sample population, further investigation is needed.

Neutropenia may be caused by other anticancer drugs such as 5‐fluorouracil or oxaliplatin. Neutropenia was also reported after FOLFOX therapy for colorectal cancer (35%) and gemcitabine plus nab‐paclitaxel therapy for pancreatic cancer (38%).^54^, ^55^ However, there was no significant correlation between *R3HCC1* genotypes and the incidence of severe neutropenia in patients treated with FOLFOX or gemcitabine plus nab‐paclitaxel (Table 6). This suggests that the *R3HCC1* genotype may be more strongly associated with severe neutropenia in irinotecan‐containing regimens than in irinotecan‐free regimens.

The limitations of this study include the small number of samples and the low frequency of the G allele of rs2272761. Therefore, the relationship between *R3HCC1* gene polymorphism and neutropenia needs to be further validated using a larger sample. In this study, we focused on neutropenia as an irinotecan‐related toxicity but did not examine other adverse effects, such as diarrhea, as they were not quantitative indicators and the incidence was too low for statistical analysis (less than 5% of patients developed grade 3 diarrhea). Although leukopenia as a quantitative indicator can also be found as an irinotecan‐related adverse effect, the analysis in this study focused on neutropenia, because neutropenia occurred more frequently than leukopenia, and leukopenia of grade 3 or higher occurred simultaneously with severe neutropenia (>grade 3). In addition, biomarkers that correlate with 5‐fluorouracil‐ and oxaliplatin‐related adverse effects would enable more precise anticancer therapy in triplet chemotherapy, such as FOLFOXIRI therapy and modified FOLFIRINOX therapy.

In conclusion, we suggest that *R3HCC1* gene polymorphism (c.919G > A, rs2272761) may be a useful predictive biomarker for severe neutropenia associated with irinotecan‐containing chemotherapy in patients with colorectal and pancreatic cancer.

## AUTHOR CONTRIBUTIONS

**Kou Kanesada:** Data curation (equal); investigation (lead); writing – original draft (lead). **Ryouichi Tsunedomi:** Conceptualization (lead); data curation (equal); formal analysis (equal); project administration (supporting); supervision (equal); writing – original draft (supporting); writing – review and editing (lead). **Shoichi Hazama:** Data curation (equal); resources (equal); supervision (equal). **Hiroyuki Ogihara:** Formal analysis (equal). **Yoshihiko Hamamoto:** Formal analysis (equal). **Yoshitaro Shindo:** Data curation (equal); resources (equal). **Hiroto Matsui:** Data curation (equal); resources (equal). **Yukio Tokumitsu:** Data curation (equal); resources (equal). **Michihisa Iida:** Data curation (equal); resources (equal). **Shin Yoshida:** Data curation (equal); resources (equal). **Nobuaki Suzuki:** Data curation (equal); funding acquisition (supporting); resources (equal). **Shigeru Takeda:** Data curation (equal); funding acquisition (supporting); resources (equal). **Tatsuya Ioka:** Data curation (equal); resources (equal). **Hiroaki Nagano:** Conceptualization (supporting); funding acquisition (supporting); project administration (lead); writing – review and editing (supporting).

## FUNDING INFORMATION

This work was partly supported by JSPS KAKENHI grant numbers 21 K08799, 22 K08849, and by the Japanese Foundation for Multidisciplinary Treatment of Cancer (JFMC), Japan.

## CONFLICT OF INTEREST

The authors have no conflicts of interest.

## APPROVAL OF THE RESEARCH PROTOCOL BY AN INSTITUTIONAL REVIEWER BOARD

The study protocols were approved by the Institutional Review Board of Yamaguchi University Graduate School of Medicine (H26‐044, H28‐171, 2020–127, and 2020–165).

## INFORMED CONSENT

Written informed consent was obtained from all patients.

## REGISTRY AND THE REGISTRATION NO. OF THE STUDY/TRIAL

UMIN00042746.

## ANIMAL STUDIES

N/A.

## Supporting information


Table S1

## Data Availability

The data that support the findings of this study are available from the corresponding author upon reasonable request.
