# Supplementary material for: Association between a single nucleotide polymorphism in the R3HCC1 gene and irinotecan toxicity
Source: Cancer Med. 2022 Oct 29;12(4):4294–305. doi: 10.1002/cam4.5299 (PMC9972014; doi:10.1002/cam4.5299)
Supplement: Supplementary file 1 — Table S1 [file CAM4-12-4294-s001.docx]

**Table S1** Relationship between *UGT1A* and toxicity in validation samples

|  | Toxicity | | |  |  |
| --- | --- | --- | --- | --- | --- |
| Genotypes | Yes | No | (% of Yes) | *P* value* | Odds ratio** |
| *UGT1A1*6* |  |  |  |  |  |
| G/G | 18 | 41 | (30.5) | - | 2.28 (*P* = 0.075) |
| G/A | 16 | 16 | (50.0) |  |  |
| A/A | 0 | 0 | - |  |  |
| *UGT1A1*28* |  |  |  |  |  |
| TA6/TA6 | 28 | 45 | (38.4) | - | 0.73 (*P* = 0.777) |
| TA6/TA7 | 5 | 11 | (31.3) |  |  |
| TA7/TA7 | 0 | 0 | - |  |  |
| *UGT1A1*60* |  |  |  |  |  |
| T/T | 17 | 31 | (35.4) | 0.591 | T/T, T/G vs G/G |
| T/G | 15 | 24 | (38.5) |  | 1.72 (*P* = 0.628) |
| G/G | 2 | 2 | (50.0) |  |  |
| *UGT1A7* (c.387) |  |  |  |  |  |
| T /T | 5 | 21 | (19.2) | 0.020 | T /T vs T /G, G/G |
| T /G | 23 | 31 | (42.6) |  | 3.36 (*P* = 0.031) |
| G /G | 6 | 5 | (54.5) |  |  |
| *UGT1A7* (c.-57) |  |  |  |  |  |
| T/T | 11 | 36 | (23.4) | 0.036 | T/T vs T/G, G/G |
| T/G | 23 | 18 | (56.1) |  | 3.58 (*P* = 0.005) |
| G/G | 0 | 3 | (0.0) |  |  |
| *UGT1A9*1b* |  |  |  |  |  |
| T10/T10 | 7 | 9 | (43.8) | 0.046 | T/T, T/G vs G/G |
| T10/T9 | 24 | 30 | (44.4) |  | 0.21 (*P* = 0.019) |
| T9/T9 | 3 | 18 | (14.3) |  |  |

* Using the Cochran–Armitage trend test. ** Using the Fisher’s exact test.
